# Maize Straw as a Valuable Energetic Material for Biogas Plant Feeding

**DOI:** 10.3390/ma12233848

**Published:** 2019-11-22

**Authors:** Jakub Mazurkiewicz, Andrzej Marczuk, Patrycja Pochwatka, Sebastian Kujawa

**Affiliations:** 1 Institute of Biosystems Engineering, Poznan University of Life Sciences, Wojska Polskiego 50, Poznań 60–627, Poland; jakub.mazurkiewicz@up.poznan.pl (J.M.); sebastian.kujawa@up.poznan.pl (S.K.); 2 Department of Agricultural, Forestry and Transport Machines, University of Life Sciences in Lublin, Głęboka 28, Lublin 20–612, Poland; andrzej.marczuk@up.lublin.pl; 3 Department of Environmental Engineering and Geodesy, University of Life Sciences in Lublin, Leszczyńskiego 7, Lublin 20–069, Poland

**Keywords:** maize straw, corn stover, methane production, biogas, substrate

## Abstract

Maize has great potential, especially as a substrate for biofuels production. The aim of this paper is to analyze the possibility of usage in methane fermentation maize straw harvested in different weather conditions, which had an influence on different physical parameters, mainly the dry mass content. The research has shown that maize straw harvested in Central-Eastern Europe can have a broad spectrum of dry mass content, which is related to diverse weather conditions during autumn. However, independently from moisture content, maize straw can be a good (for more wet material) or very good (for more dried straw) substrate for the biogas plant. With the methane productivity reaching 201–207 m^3^/Mg of fresh mass, this material is a significantly better substrate than that typically used in Europe maize silage (approximately 105 m^3^/Mg FM). It was noted that the retention time for maize straw (36–42 days) is longer than in the case of maize silage (less than 30 days). However, this difference is quite small and can be accepted by the biogas plant operators.

## 1. Introduction

Maize straw (also known as corn stover) is one of the most common materials produced in agronomy. However, especially in Europe, the utilization of this material has been rather weak. For most farmers, its usage was limited to cutting during harvesting and then ploughing [1]. Removing crop residues from the field for commercial biofuel must balance preventing soil erosion, maintaining soil organic matter, and maintaining or increasing productivity [2,3]. 

In the last decade, global maize grain production has increased by around 40%, and now amounts to almost 1100 million tonnes [4]. In 2017, EU maize grain production was over 70 million tonnes [5], and for comparison in Beijing in 2012 alone, 1.20 million tonnes maize straw was produced [6], and all over China more than 2.7 × 10^8^ tonnes has been produced annually [7]. Such a high production of maize grain causes crop residues, such as leaves, stalks, husks, and cobs, which can constitute up to 50% of the dry matter yield of whole maize plants [8,9]. The fractions mentioned above have different chemical compositions, structure, and fiber properties [10,11], harvesting times, and even topography or soil types [12,13]. For example, sufficient storage as a result of ensiling operations enables 1.1%–2.2% reduced loss of organic matter compared to in open-air storage (63.1%) [7].

For 1 kg of harvested maize grains (expressed in dry matter), the total mass of maize parts (leaves, cobs, husks, and stalks) is approximatively 1.01 kg of dry matter. Regardless of the characteristics mentioned above, maize straw is a valuable energy resource [9,13,14]. Analysis conducted by scientists from the Poznan University of Life Sciences has shown that annual maize straw production in Poland may reach 4 million tonnes in the near future [15]. 

### 1.1. Direct Combustion of Maize Straw

One way of obtaining energy from maize straw is through direct combustion. The calorific values of the maize straw range from 17.65 to 18.6 MJ/kg of dry matter [9,16]. This is the general value given without specifying the proportion of individual fractions, age, moisture content, or variety. The variability of gross energy of different straw fractions during aging was very different. In extreme cases, the differences were even 50% [16]. It is emphasized that such large fluctuations in values were caused by sample heterogeneity and/or lack of consistency in calorimetric procedures, suggesting the adoption of mean values in the range of 16.7–20.9 MJ/kg [16]. In addition, they indicate that the energy content of different maize fractions remains fairly constant over time and between individual plants, and therefore they suggest that in the case of combustion, there is no significant difference regarding what fraction and at what moment the plants were harvested. When comparing maize straw with other biomass fuels, attention should also be paid to the content of ash between 4% and 6.8% (particularly with a high concentration of silica that is more than 34% and potassium levels that are higher than 30%), large amounts of nitrogen (0.6% N), sulfur (0.09% S), and chlorine (0.36% Cl) [9]. These are significantly higher values compared to premium wood pellets [17,18], which must contain less than 0.3% N, 0.05% S, and 0.03% Cl [10].

Research results from other scientists also show quite large variations due to the maize straw fraction and early or late harvest time. The ash content measured was: for husk—2.1%, for cob—1.1% (both for late straw harvest), for leaves from 2.4% to 3.4%, and for stems from 6.0% to 7.3% for the early and late fraction, respectively [13].

Such an ash content can significantly reduce the efficiency of heat exchange as a result of slagging when the combustion temperature is low, and chlorine compounds can accelerate boiler corrosion [19]. From a practical point of view, maize straw showed good caloric potential, but the fuel produced from it would have to be concentrated, and the current combustion technique would require a lot of work due to the large amounts of ash.

### 1.2. Bioethanol Production

Another way to use the energy stored in maize straw is, for example, the production of bioethanol [18]. In particular, the bioconversion of maize straw has been a subject of interest, because this residue is available in large quantities and at low cost [20,21]. 

Concern over maize straw removal argued that some must be left on the field to sustain the humic content of the soil and maintain soil productivity, and there may also be constraints in supply and its distribution [3]. In spite of this, maize straw has both economic and environmental potential for the production of bioethanol as a shift from gasoline, and thereby provides a substitute for fossil fuel [22,23].

Maize straw, as a typical lignocellulosic raw material, contains lignin, cellulose, and hemicellulose, which together form a complex polymer structure that limits reaction media or enzymes to close contact with cellulose; in this way, maize straw is not easily converted into bioethanol [24]. For practical reasons, it is characterized by low mass and energy density, is resistant to degradation, and pre-treatment is necessary. Simultaneous saccharification and co-fermentation were developed for the production of cellulosic ethanol, but the concept was misdefined because saccharification and co-fermentation are by no means simultaneous [23]. Lignin is not reactive, which not only takes up the reactor spaces during the enzymatic hydrolysis of the cellulosic component and subsequent fermentation of ethanol, but also requires additional mixing. This significantly impedes the high solid load of lignocellulosic biomass and ethanol titles, which consequently increases the energy consumption for ethanol distillation and discharge stillage, and is another challenge for the production of cellulosic ethanol [23].

Based on 144 data sets that were sufficiently consistent and detailed to take into account the current state of the art of converting maize straw to bioethanol, researchers found that the best technological configurations produced 19%–22% ethanol (dry weight), while the technological configuration with the lowest efficiency produced only 11% ethanol [24]. Besides bioethanol production, it was observed in the review that all technological configurations produced large flows of solid and liquid residues, accounting for 55%–75% of the carbon in the maize straw. Bioethanol from residual maize straw could contribute to lowering CO_2_ loads within the transport sector, if used as an amendment to gasoline. The “best-practice”, defined as the top 15% cumulative probability with respect to the Global Warming Potential (GWP), suggests that technologies based on steam explosion and ammonia-based pre-treatment statistically appear to be the most promising. These technologies could contribute, with residue energy recovery, to GWP savings of 850–1050 kg CO_2eq_/Mg dry maize straw solids and produce 178–216 kg of bioethanol [25]. Other analyzes, which are extensive but fairly general, indicate that the greenhouse gas (GHG) savings from cellulosic ethanol in the decentralized system compared to gasoline are 3.35–4.84 Tg CO_2_ per year [26]. 

An interesting development of maize straw processing used for energy production is the method in which the water produced during the pre-treatment stage of bioethanol production was recovered and reused as a co-substrate for the anaerobic digestion. Such water contains high concentrations of lignocellulolytic enzymes, which once added to the fermentation chamber, improved the kinetics of methane production. Compared to digestion in the absence of these additional enzymes, the final methane yield increased by 42%, with a 67% reduction in lag-phase time and a nearly 30% increase in the methane production rate [27].

### 1.3. Economic and Energy Production Potential

Generally, the cost of agricultural residues for European conditions can be estimated at USD 1–8 GJ. There are large differences in biomass production potential and costs between European regions, with 280 regions (NUTS2). Regions that are distinguished by high potential and low costs include a large part of Poland, the Baltic States, Romania, Bulgaria, and Ukraine. In Western Europe, France, Spain, and Italy are moderately attractive, given the high potential low cost criterion [28].

Research has shown that burning a maize in a furnace can replace a significant amount of fossil fuel traditionally used to dry maize grain in many locations. According to analysis, the use of maize straw as the main source of energy for drying grain would be profitable both for a small scale 8.9 Mg/h dryer with an average of 0.7 MW of heating power, and on a large scale 73 Mg/h dryer, with an average of 6.3 MW [9]. The same authors stated that based on 2004 prices, small-scale drying would have a 14–year payback period with harvest and transport prices of USD 25 Mg/DM (dry matter), and even 8 years if it was dried at higher prices such as USD 45 Mg/DM (due to higher transport costs to the destination) due to a clear impact of the scale effect [9].

Using maize would provide an alternative to maize straw for biochar production. This form of solid biofuel allows for the reduction of transport costs and investments in fuel storage. In addition, the pyrolysis of biomass pellets has obvious advantages, such as lower equipment costs, a simple equipment system, and high production efficiency [29]. Biochar pellets can reduce the costs and problems associated with the transport and storage of biomass due to its stable quality, high resistance to weather conditions, and suitability for long-term storage. For these reasons, some scientists argue that biomass pellets should be used to a much greater extent in the energy sector. At the same time, they add that the popularization and use of pellets is extremely expensive (the cost of pellets is about USD 75/Mg, while the raw material is around USD 3.2/Mg) [29,30].

Based on information from the Slovak Republic, it can be concluded that maize straw in the form of briquettes and pellets has a high energy potential, which is comparable with currently used materials for the production of briquettes and pellets. The authors highly recommended using all current waste materials, as well as all types of waste materials (especially biological) to preserve the main key factors of proper waste management [31].

Newer analyses of the Polish market indicate instead that the transformation of corn straw by methane fermentation to obtain electricity is more profitable than the production of solid fuel from it. On the one hand, the total amount of energy contained in biogas produced from 1 Mg of maize straw is 9.74 GJ, and this value is slightly lower than the net energy that can be used to burn briquettes [32]. It should be emphasized that over 97% of European biogas plants use biogas for the production of electricity and heat in cogeneration units. Thus, the actual energy value from the energy transformation of maize straw by burning the produced biogas is 0.97 MWh of electricity and 4.21 heat. About 15% of energy is lost, mainly in the form of heat. On the other hand, economic analysis of briquette production shows a much lower profit compared to the consumption of corn straw as a substrate for biogas production. This is mainly due to the fact that the price of heat in Poland is several times lower compared to the price of electricity (in Poland: electric energy price = 150 USD/MWh and heat energy price = 12.5 USD/GJ) [32]. As a consequence, even the conversion of corn straw into solid biofuel (briquettes) can generate more energy from 1 Mg of biomass, thereby providing thermal energy which is cheaper than electricity. The uncertain future of straw use in power plants can be seen in Great Britain. There, changes in energy tariffs caused a decrease in interest from the entire industry in the production of solid fuels from maize straw, as a result of which several plans to build straw granulation plants were suspended [33].

Newer studies analyzing the costs of acquiring and processing maize straw into biogas in more detail indicate that they differ depending on several factors. Maize straw harvesting and storage technology has a significant impact on economic results. Research has shown that collecting corn maize with the help of a self-loading wagon equipped with a cutting system and storing it in a field prism is the most profitable option. The cost of this technology is EUR 12.5 per Mg DM. The cost of harvesting corn straw using a chopper and storage in a flexible silo was the highest, at EUR 126.9 per Mg DM. The test results can be used to estimate the profitability of harvesting corn straw for energy production and other industrial purposes [34].

The use of maize straw for energy purposes is economically and energetically justified. However, the relatively high humidity of this material in its fresh state means that the possibilities of its rational use for heating purposes are strictly limited [35,36,37,38]. Therefore, maize straw has far fewer potential buyers, and hence a significantly lower price than straw from cereal ears and even from rapeseed production [36]. However, in the case of methane fermentation, it can be one of the cheaper and more easily available agricultural substrates [39,40,41].

For example, the total capital investment for the annual volume of ethanol in a decentralized biorefinery is from USD 0.71/dm^3^ to USD 1.15/dm^3^, and the total capital investment for the annual volume of ethanol production in a centralized biorefinery is USD 1.98/dm^3^ [26]. The production costs of renewable diesel is USD 1.19 /dm^3^ for the corn stover hydrogen production. The net energy ratio (NER) of the process, which is the ratio of the energy content of the output product to fossil fuel inputs, was calculated to be 1.5 [35]. An interesting comparison was established based on a similitude of the efficiency of algae and maize straw use. Algae are able to achieve the highest surface biomass production rates of 120 Mg/ha·a, ten times higher than maize, but at the same time with the highest costs and the lowest rate of return, in addition to more than three times higher CO_2_ emissions levels [42].

It can be concluded from many studies to date that the use of lignocellulosic ethanol (as in the case of maize straw) on a large scale would require more sustainable and cost-effective agricultural practices, since ethanol production is highly dependent on the cost of the raw material, as well as the development of advanced ethanol biorefinery technologies [43,44]. Generally, the price of raw material has a big impact on raw material supply chains, although not directly. Namely, the higher raw material price has no major impact on the final ethanol sales price, but higher raw material prices significantly increase the amount of ethanol produced. Farmers significantly increase the supply of cellulose biomass at higher prices of raw materials, then the reduction of the final price is primarily due to transport costs and the effect of scale that larger biorefineries have, thus reducing the ethanol sales price [44,45]. Currently, only single studies can be found that suggests that producing bioethanol at smaller local points is economically profitable [26]. 

### 1.4. Biogas Production

There are almost 20,000 biogas plants working in Europe. Except for in Denmark, Switzerland, and Sweden, the most popular substrate used for feeding agricultural biogas plants is maize silage produced from whole plants. This substrate maintains stable biogas production on a good level (approximately 105 m^3^ CH_4_/Mg of Fresh Mass) and is easy to harvest and store. With a high level of subsidies in many EU countries, this substrate (despite being quite expensive) has guaranteed good rentability for most of the agricultural biogas plants. That was a major reason for the huge development of maize cultivation for biogas plants feeding in places like Germany where over 10% of the agricultural land surface is used for silage production to cover biogas sector needs. 

However, during last decade, many European countries have introduced limitations for Renewable Energy Sources subsidies. This also affected the biogas sector. From the other side, maize silage prices experienced significant growth, which reduced biogas plant rentability. That is why many biogas plant operators started to look for alternative substrates which can be easy to get, are inexpensive, and have good methane productivity. Maize straw can be one of these substrates, which has also been confirmed by Chinese experiences [7,46].

The aim of this paper is to analyze the possibility of using maize straw harvested in different weather conditions in a methane fermentation process, and as a consequence having different physical parameters (mainly different dry mass content). It has to be mentioned that weather conditions in Central-Eastern Europe during autumn (the time of maize harvesting for grain production) are very unstable. That is why maize straw parameters are deeply dependent on weather conditions.

## 2. Materials and Methods

The analyzed material (different maize straws) was collected from 4 farms situated in Western and Eastern Poland. Straw was collected manually after a grain harvest (average 10 kg for each sample), directly from the field, and then transported to the Ecotechnologies Laboratory (at the Poznan University of Life Sciences, PULS) for basic analyses and biogas production tests using methane fermentation. Straw was collected under different weather conditions, which should have a strong influence on its physical characteristics.

Collected materials (different maize straws) were classified within dry matter content. That is why the material names (each maize straw, MS) contain dry matter (TS) content. The analyzed straws names were: MS45, MS55, MS78, and MS89 (numbers mean the initial dry matter content is expressed based on the percentage of dry matter). 

### 2.1. Physical Analysis

Maize samples initially were analyzed for dry matter content (Total Solids—TS, within the Polish Standard PN-75 C-04616/01 [47]). The standard procedure contains drying samples (in 3 repetitions) heated to 105 °C over 24 h. Organic matter (Volatile Solids, VS) content was analyzed within the standard PN-Z-15011-3 [48] by combustion of dry samples (in 3 repetitions) at 525 °C over 3 h. The samples were also analyzed for pH (PN-90 C-04540/01 [49]) and conductivity (PN-EN 27888: 1999 [50]).

It has to be underlined that data about TS and VS are indispensable for starting fermentation tests and then, for calculating the methane and biogas production efficiency of each analyzed straw into the units m^3^/Mg FM (Fresh Matter), m^3^/Mg TS, and m^3^/Mg VS. While results expressed in CH_4_ production in m^3^ per VS are most commonly used in research papers to compare the efficiency of each substrate, in real biogas plant exploitation the most important parameter is substrate methane efficiency expressed in CH_4_ by Mg of FM (Fresh Matter). 

### 2.2. Methane Fermentation Tests

**Figure 1:**
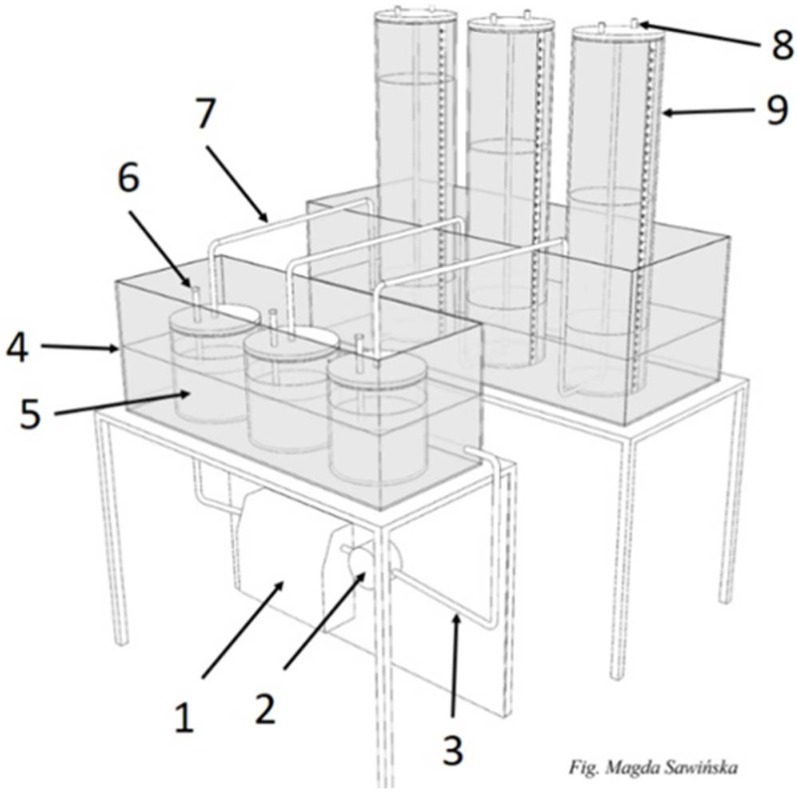
Scheme of a fermenter for biogas production research (3-chamber section): 1—water heater with temperature regulator, 2—water pump, 3—insulated conductors of calefaction liquid, 4—water coat, 5—fermenter with charge capacity 2 dm^3^, 6—sampling tubes, 7—biogas transporting tube, 8 - gas sampling valve, 9—biogas volume-scale reservoir [53].

Biogas production analysis was conducted at Ecotechnologies Laboratory, the biggest Polish biogas unit. The laboratory uses the German’s standards: DIN 38 414/S8 [51] and VDI 4630 [52]. This laboratory received the Proficiency Test Biogas certificate for a framework of international tests organized by German KTBL in 2017, as it was the first Polish biogas laboratory which provided high-quality research in the area of methane fermentation. The experiment of straw samples anaerobic digestion has been conducted in a special 21-chambers fermenter. The simple version of a 3-chambers section fermenter is shown in Figure 1.

The fermentation procedure of the anaerobic digestion tests proceeded in glass fermenters with volumes of 2 dm^3^ each. The analyzed substrates were put inside each reactor with the weight-related to the VS content and then were mixed with inoculum standardized with DIN 38 414/S8 and VDI 4630 standards. Reactors were flushed with nitrogen gas in order to remove air and immediately create anaerobic conditions (oxygen is an inhibitor for methanogenic bacteria). All samples were fermented in 3 replications, with final results representing an average value of those measurements. 

The biogas production measurements were taken every 24 h by connecting gas reservoirs (9) (Figure 1) through sampling valve (8) with gas analyzer Geotech GA5000. This analyzer possesses quality certifications like ATEX II 2G Ex ib IIA T1 Gb (Ta = −10 °C to + 50 °C), IECEx, CSA, and the calibration certificate UKAS ISO 17025. The measurement ranges for the Geotech analyzer are: O_2_ 0–25%, CO_2_ 0–100%, CH_4_ 0–100%, NH_3_ 0–1000 ppm, and H_2_S 0–10 000 ppm. GA5000 was usually calibrated once a week using calibration gases (from the Air Product company) in the concentrations: 65% for CH_4_, 35% for CO_2_ (in one mixture), 500 ppm H_2_S, 100 ppm NH_3_, and O_2_ using synthetic air.

The end of the methane fermentation experiment (within DIN 38 414/S8 standard) was the moment when daily biogas production was less than 1% of the total volume of obtained biogas. The efficiency of examined materials for biogas and methane productivities can be expressed using low, good, or very good levels. In described research, this criterion concerns chemical energy production from maize straw and is expressed in the amount of methane produced from the mass unit (m^3^ CH_4_/Mg). Since the most common substrate used in European biogas plants is maize silage with the methane productivity 105 m^3^/Mg, all straws was analyzed under the criteria “low quality substrate”, “good substrate” (like maize silage), and “very good substrate”. 

## 3. Results

### 3.1. Physical Analysis

The analysis of dry matter (TS) and organic dry matter (VS) is shown in Table 1. 

The analysis of TS shows a significant difference between tested materials because dry matter content varies from 45% to 89%. This vast difference is related to very unstable weather conditions (different rainfall and temperatures) during the autumn period in Poland and as a consequence strongly influences the viability of maize straw storage technologies. It has to be underlined that for more dry materials like in case of MS78 and MS89, maize straw can be stored in the pressed form (bales). However, a material with higher moisture like MS45 and MS55 should be stored over a longer time as silage because the additional tests of more wet straw storage in bales increase their temperature and start the quasi composting process. This phenomena strongly reduces the energetic potential of maize straw through non-controlled losses of heat from worm bales.

Considerably smaller differences between analyzed materials were found in the case of organic matter content (VS). The highest VS content in the straw with higher moisture content could be related with the “cleaning” effect of the rains that occurred before and during the harvest. The straws harvested during dry weather contained more soil dust which increased mineral matter content up to over 10% in case of MS78 and MS89 (Table 1). In contrast, the most wet straw (MS45) harvested under rainy weather only had 2.41% mineral matter and the highest organic matter content (97.59%).

### 3.2. Fermentation Results

**Figure 2:**
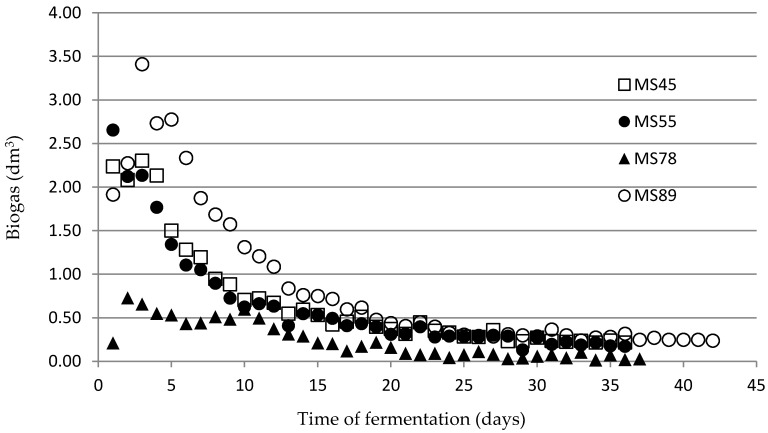
Production of biogas during materials fermentation process (daily measurements) for all materials.

The results of biogas production are presented on Figure 2. It is important to underline that results of biogas production (expressed in dm^3^) cannot be directly compared at this stage between tested materials because of the different initial fresh mass used for tests. The German standard DIN 38 414/S8 needs to use the amount of sample calculated in organic dry matter (VS), so the initial fresh matter amount is usually different. The final biogas and methane productivity results (after calculations required by the DIN standard) are presented in Table 2.

The results of the biogas efficiency test show that the most intensive gas production occurred during the first 10 days of the process. The fermentation time was almost the same for 3 analyzed materials (36–37 days), while only the driest straw (MS89) had the process occur for 42 days. This was probably related to having the highest concentration of hardly fermentable fibers and can be a subject of future study.

**Figure 3:**
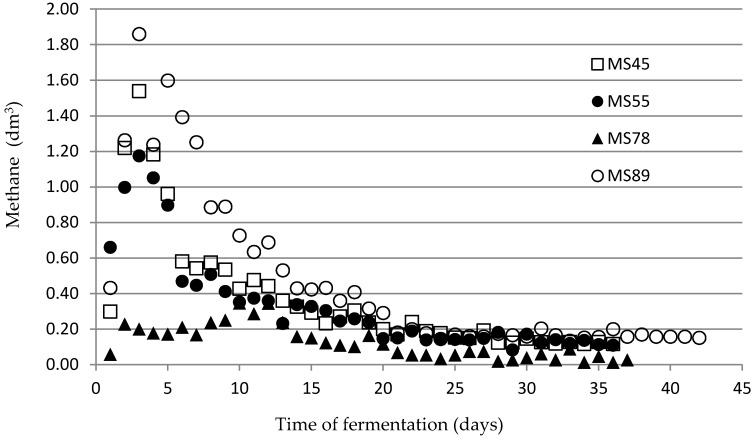
Methane production during fermentation process (daily measurements) for all materials.

However, the methane production results are shown in Figure 3. 

It should be underlined that methane (contained in biogas) is the crucial parameter of estimated substrates. The biogas contains mainly methane (the fuel for the co-generation unit) and carbon dioxide (the ballast). Similarly to for biogas production, methane generation also occurred mainly during the first 10–13 days. After the 20th day, the methane production stayed low for all tested materials. 

Using the procedures described in the standard DIN 38 414/S8, the calculations were made in order to present the biogas and methane production results from analyzed straws, expressed in the unit which is the most often used by specialists from the biogas sector (m^3^ of methane from Mg of the substrate). Those calculations also take into account the production from inoculum used for initializing the fermentation process. The results of biogas and methane productivity from tested straws are shown in Table 2.

The data content in Table 2 shows that methane concentration was very similar (approximately 49%–50%) for all tested materials. The CH_4_ production from analyzed material was very different (from 99 to 207 m^3^/Mg) in the calculation based on the fresh matter, but this is logical because a higher concentration of dry matter correlates with higher methane production. Typical maize silage from whole plants can reach the methane’s productivity level at approximatively 105 m^3^/Mg. The results obtained for wet straws (MS45 and MS55) are a little bit lower compared to maize silage, but in case of more dry straws, the productivity is almost 2 times higher.

However, when looking at CH_4_ production expressed in m^3^/Mg of VS, the differences in productivity level are much lower (194–288 m^3^/Mg VS). This means that independently of maize straw moisture, this material is still attractive as a substrate for a biogas plant. 

**Figure 4:**
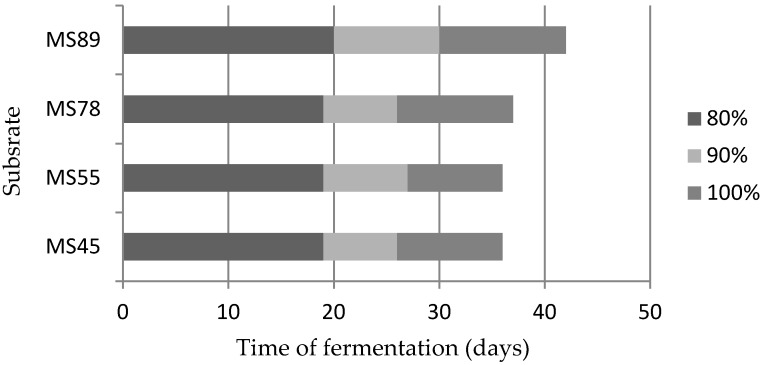
Time required 80%, 90%, and 100% total methane production from analyzed maize straws.

From a biogas plant manager’s point of view, the time of substrate total fermentation is very important because with shorter biogas production process more substrate can be treated in fermenters with the same volume. Figure 4 shows the time needed for complete fermentation as well as for 80% and 90% methane production levels.

The time required for complete fermentation of analyzed straws is between 36 and 42 days. However, 90% of methane production occurred before 30 days of the process. This means that a shorter process in the main fermenters (up to 30 days) can generate most of the energetic gas. The rest can be obtained in the digested tank, if it is covered by a hermetic cover and is equipped with a heating system. It has to be underlined that the time needed for complete methanization of all analyzed straws was longer than for a typical maize silage fermentation, which is usually less than 30 days [53]. However, maize straw retention time (36–42 days) is still acceptable for biogas plant operators and is almost 3 times shorter than for a typical straw fermentation (105–120 days) in NaWaRo technology, which is the most frequently used technology type in Europe. 

## 4. Discussion

Maize straw is a material produced in huge quantities on a worldwide scale. However, compared to cereals straw, this material is rarely used in Europe and usually stays on the field after harvest as an organic fertilizer [54]. In this study, we have shown that maize straw can be a valuable substrate for a biogas plant, especially if the material has a higher dry matter content. In this case, the methane productivity (201–207 m^3^/Mg FM in the case of MS78 and MS89) is clearly higher compared to maize silage from whole plants (approximately 105 m^3^/Mg FM), which is the most popular substrate in European biogas plants [46,55,56,57].

One of the problems related to maize straw utilization is farmer fear of proper methods for its harvest and storage. However, within the PULS research project conducted in 2010–2013 by the scientists from Institute of Biosystems Engineering, 6 harvest technologies and 3 maize straw storage technologies were developed. This and the research that followed have shown the large possibilities of maize straw harvesting and utilization for biogas plants feeding in the form of silage, but also directly as dry material stored in bales [34,57,58]. The farmers should therefore be able to know the moisture content of harvested maize straw. However, this parameter should be the key element for making a decision about the maize straw storage technology they should use: either using compressed bales or a silage process under the chopped straw form. Taking into account the possible technologies of maize straw management, we think that weather conditions can be important during harvesting time would not prevent maize straw utilization as a valuable substrate for biogas plant. In this case, the farmers should measure the dry matter content/moisture of maize straw before harvest and then choose the best technology for its storage (in bales or in silos).

The significant advantage of maize straw (instead of maize silage) usage for biogas is the exclusion of the conflict between food and biofuels production. Maize straw is the additional biomass on the field from grain production. Its utilization for biogas production helps to manage the chemical energy contained in straw organic matter, which under typical scenarios is used by microorganisms proceeding its decomposition in soil. It is also important the macro and microelements contained in maize straw (except carbon) are not lost in the case of using the straw in a biogas plant because, after fermentation, those minerals came back to the soil with digestate. 

There are some ideas to use the maize straw for bioethanol production, solid biofuels, or as a structural substrate for composting in windrows [32,38,58]. However after taking energetic, economic, and agricultural into account aspects, the usage of maize straw as a substrate for biogas plants seems to be the most promising option for the near future. 

## 5. Conclusions

The research described in this paper let us reach the following conclusions:

1. Maize straw harvested in Central-Eastern Europe can have a broad spectrum of dry mass content, which is related to diverse weather conditions during autumn.
2. Independently from moisture content, maize straw can be good (for more wet material) or very good (for more dried straw) substrate for the biogas plant. With the methane productivity reaching 201–207 m^3^/Mg of fresh mass, this material is significantly better substrate than that typically used in Europe maize silage (approximately 105 m^3^/Mg FM).
3. The retention time for maize straw (36–42 days) is longer than for maize silage (less than 30 days). However, this difference is quite small and can be accommodated by the biogas plant operators.

## Figures and Tables

**Table 1:** The initial parameters of the tested material (maize straws) used for fermentation.

Substrate	TS (% FM)	VS (% TS)
MS45	44.77	97.59
MS55	54.68	93.41
MS78	77.80	89.78
MS89	89.46	89.50

**Table 2:** The biogas and methane production from tested materials.

Substrate	CH_4_ Content (%)	CH_4_ m^3^/Mg FM	Biogas m^3^/Mg FM	CH_4_ m^3^/Mg TS	Biogas m^3^/Mg TS	CH_4_ m^3^/Mg VS	Biogas m^3^/Mg VS
MS45	49.51	103.39	206.47	230.93	461.17	236.64	472.56
MS55	48.97	98.95	199.94	180.96	366.09	193.73	391.45
MS78	50.17	201.12	400.90	258.52	515.29	287.94	573.95
MS89	50.26	206.78	411.43	231.14	459.90	258.26	513.86
